# Cross-Cycled Uplink Resource Allocation over NB-IoT

**DOI:** 10.3390/s21237948

**Published:** 2021-11-28

**Authors:** Ya-Ju Yu, Yu-Hsiang Huang, Yuan-Yao Shih

**Affiliations:** 1 Department of Computer Science and Information Engineering, National University of Kaohsiung, Kaohsiung 811, Taiwan; yjyu@nuk.edu.tw (Y.-J.Y.); a1075503@mail.nuk.edu.tw (Y.-H.H.); 2 Department of Communications Engineering, National Chung Cheng University, Chiayi 621, Taiwan

**Keywords:** NB-IoT, uplink resource allocation, cross-cycle, cellular networks, massive connections

## Abstract

Before each user equipment (UE) can send data using the narrowband physical uplink shared channel (NPUSCH), each UE should periodically monitor a search space in the narrowband physical downlink control channel (NPDCCH) to decode a downlink control indicator (DCI) over narrowband Internet of Things (NB-IoT). This monitoring period, called the NPDCCH period in NB-IoT, can be flexibly adjusted for UEs with different channel qualities. However, because low-cost NB-IoT UEs operate in the half-duplex mode, they cannot monitor search spaces in NPDCCHs and transmit data in the NPUSCH simultaneously. Thus, as we observed, a percentage of uplink subframes will be wasted when UEs monitor search spaces in NPDCCHs, and the wasted percentage is higher when the monitored period is shorter. In this paper, to address this issue, we formulate the cross-cycled resource allocation problem to reduce the consumed subframes while satisfying the uplink data requirement of each UE. We then propose a cross-cycled uplink resource allocation algorithm to efficiently use the originally unusable NPUSCH subframes to increase resource utilization. Compared with the two resource allocation algorithms, the simulation results verify our motivation of using the cross-cycled radio resources to achieve massive connections over NB-IoT, especially for UEs with high channel qualities. The results also showcase the efficiency of the proposed algorithm, which can be flexibly applied for more different NPDCCH periods.

## 1. Introduction

The primary purpose of narrowband Internet of Things (NB-IoT), standardized by the 3rd Generation Partnership Project (3GPP), is to serve massive low-cost UEs efficiently [1]. As forecasted by Cisco, the number of IoT connections will be 14.7 billion and will account for about half of the global connections by 2023 [2]. Moreover, by 2026, NB-IoT and LTE-M technologies are expected to serve approximately 45% of all cellular IoT connections [3]. Therefore, efficiently utilizing the radio resources of NB-IoT networks is essential for achieving a large number of connections.

**Figure 1:**
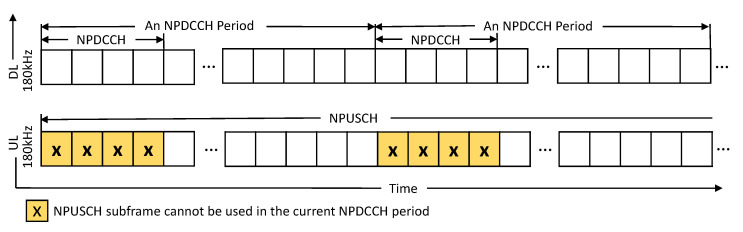
Waste uplink subframes in NB-IoT.

To use the radio resources in NB-IoT networks, before each user equipment (UE) can send data, each UE must periodically monitor a search space in the narrowband physical downlink control channel (NPDCCH) to decode a downlink control indicator (DCI). A DCI is used to carry the uplink parameters for a UE so that the UE can know how to use the narrowband uplink shared channel (NPUSCH) subframes for transmitting data. This period is called the NPDCCH period (NP) in NB-IoT and can be flexibly adjusted for UEs with different channel qualities. However, because low-cost NB-IoT UEs operate in the half-duplex mode, the UEs cannot simultaneously monitor search spaces in NPDCCHs of a downlink frequency (DL) and transmit data in the NPUSCH of an uplink frequency (UL). As a result, when the UEs periodically monitor the search space in the NPDCCH, we observe that a percentage of uplink subframes will be wasted, as shown in Figure 1. Furthermore, the wasted percentage is higher when the NPDCCH period length is shorter. To overcome this issue, we propose the concept of cross-cycled resource allocation to efficiently use the traditionally unusable NPUSCH subframes to increase resource utilization. Specifically, we allow UEs to stop monitoring their search spaces in the next NP while continuously transmitting data in the NPUSCH subframes from the current NP to the next NP without causing any interference. We explain the cross-cycled resource allocation in detail in Section 2.

Recently, there have been some studies exploring various uplink resource allocation issues in NB-IoT. Yu et al. [4] proposed a method that is divided into inner and outer loops. The inner loop adjusts the number of repetitions to ensure a transmission error rate and reduce resource consumption. The outer loop determines the modulation-coding schemes and the number of repetitions. However, this paper only considers a single tone. Besides, Elgarhy et al. [5] decomposed the problem into uplink scheduling and power allocation to maximize the throughput while maintaining as low latency as possible. Hsieh et al. [6] designed an algorithm to allocate NPDCCH and NPUSCH resources for multiple coverage enhancement (CE) levels. Mostafa et al. [7] studied the power and subcarriers allocation to maximize the number of service UEs and meet each UE’s needs.

Huang et al. [8] investigated the influence of scheduling parameters on the resource allocation problem and designed an algorithm to schedule a large number of UEs. Pei et al. [9] proposed an algorithm to control the number of UE connections to increase the probability of successful connections by ensuring that the UEs complete the random access and data transmission processes. Kodheli et al. [10] considered the use of low-Earth orbit satellites to provide the NB-IoT connectivity to on-ground UEs. Liang et al. [11] investigated the link adaptation problem and designed a heuristic algorithm to minimize the energy consumption. Finally, Yu et al. [12] designed a resource allocation algorithm that considers narrowband physical random access channels (NPRACHs) to minimize the consumed subframes. However, these works do not consider the cross-cycled allocation for NB-IoT and waste many subframes, especially when an NPDCCH period length is short.

In this paper, we study the cross-cycled uplink resource allocation problem over NB-IoT networks. Our goal is to reduce the number of consumed subframes while satisfying the data request of each UE. The contributions of this paper are summarized as follows.

- We believe this is the first paper to consider the cross-cycled resource allocation for the uplink in NB-IoT networks.
- We propose a cross-cycled uplink resource allocation algorithm that can use the subframes of the next NP without causing interference.
- We conducted experiments via simulations using realistic settings. Compared with the two resource allocation algorithms [11,12], the results verify our observation and demonstrate the efficiency of the proposed algorithm, which can reduce more subframes with a smaller NPDCCH period. Moreover, the proposed algorithm can be applied for more different NPDCCH periods.

The remainder of this paper is organized as follows. In Section 2, we describe the system model and the problem formulation. In Section 3, we explain the proposed algorithm and analyze its properties. Section 4 presents the simulation results. Finally, Section 5 concludes this paper.

## 2. System Model and Problem Formulation

### 2.1. System Model

**Figure 2:**
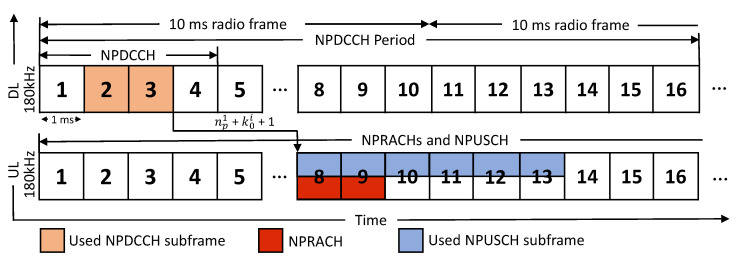
Resource allocation protocol in NB-IoT frames.

This paper considers that a cell needs to meet the uplink data requests of the NB-IoT UEs with high channel qualities, i.e., the UEs are near the cell. Figure 2 shows the NB-IoT frame structures, which adopt orthogonal frequency-division multiple access (OFDMA). The bandwidth of an uplink and a downlink channel is 180 kHz, and a radio frame is 10 ms long and contains ten subframes, each 1 ms long. In addition, an uplink subframe has 12 or 48 subcarriers, and we consider the former because it is mandatory in NB-IoT.

NPRACHs and NPUSCH share the same uplink radio resources in an uplink frequency, and devices should execute the random access procedure in NPRACHs, which can be repeated with the smallest period of 40 ms and up to 2560 ms. The period can be adjusted based on traffic loading. The subcarrier space of NPRACHs is 3.75 kHz, and this paper considers that NPRACHs are predetermined. NPUSCH is used for UEs to transmit data to the cell. A cell can provide four resource unit types occupying different subcarriers in the frequency domain and subframes in the time domain: 12 subcarriers (tones) with 1 ms, six subcarriers with 2 ms, three subcarriers with 4 ms, and a subcarrier with 8 ms. A resource unit is the smallest allocable unit. A cell should decide which NB-IoT UE uses one unit type to transmit its data. To avoid interference, each subcarrier can be used by only one UE or channel.

In NB-IoT systems, the data transmission of each UE must follow the control instructions, namely downlink control indicators, from the cell. The channel carrying DCIs is called the NPDCCH. Each UE should periodically monitor its NPDCCH, which is known as its search space, to decode a DCI. A UE should monitor a common search space before the random-access procedure and can monitor a specific search space after the random access procedure. This paper considers that the UEs have high channel qualities at the first CE level and monitor the same common search space. Nevertheless, this paper can also be applied directly to specific search spaces. As shown in Figure 2, the NPDCCH period of a UE consists of a series of NPDCCH subframes and a series of narrowband physical downlink share channel (NPDSCH) subframes. The number of NPDCCH subframes is determined by the parameter $R_{max}$, and the NPDCCH period can be set by the two parameters, $R_{max}\times G$, for a UE, where *G* is a system parameter [1] (Readers can refer to [13] for background about the NPDCCH period, signals in the frame structures, and CE level determination). Here, we assume that the UEs’ NPDCCH period (i.e., the common search space period) has been determined.

A cell should determine four parameters for a UE to use uplink radio resources: resource unit type, modulation, and coding scheme (modulation for short), scheduling delay, and resource assignment. A DCI carries these four parameters. The used unit type, modulation, signal-to-noise ratio (SNR), and amount of transmitted data will affect the required data repetition number of a UE to achieve the required transmission reliability. Therefore, some unit type and modulation combinations are not usable for a UE if the required number of repetitions is higher than the maximum repetition number. Specifically, if a UE has a better channel quality, the cell can have more combination selections of a unit type and a modulation-coding scheme for the UE. Similarly, a cell transmitting a DCI for a UE should also apply a DCI repetition number to meet the reliability.

After determining the above two parameters, the cell should allocate the other parameters for a UE: resource assignment and scheduling delay value. The resource assignment field is the number of units that do not include the repetition number assigned to a UE. Then, the resource assignment field and the modulation-coding index used can determine the number of bits (i.e., the transport block size (TBS)) the UE can transmit. The scheduling delay determines how many subframes are needed to delay the delivery of data after a UE receives a DCI. The total delayed subframes for the uplink can be computed based on

(1) $$\begin{array}{c} t_{i}^{c}=n_{p}^{c}+k_{0}^{i}+1. \end{array}$$

$k_{0}^{i}$ is *i*-th delay value, and $n_{p}^{c}$ is the last subframe in a series of NPDCCH subframes for delivering DCI *c* during the *p*-th NP [1]. Constant 1 is the changing time from DL to UL. For ease of presentation, subframe $t_{i}^{c}$ is namely *NPUSCH start subframe*.

Here we use Figure 2 as an example to explain how the unit type, resource assignment, and scheduling delay mechanisms work in uplink subframes of NB-IoT. We set the DCI repetition number for each UE as two such that $n_{p}^{1}$ is 3. If the cell selects a scheduling delay $k_{0}^{1}=4$ for a UE, the UE will transmit data from the NPUSCH subframe 3+4+1=8. Then, for the resource assignment, the cell allocates two resource units, six subcarriers with 2 ms, to the UE with a data repetition number of 3. As a result, six consecutive subframes are allocated to the UE. Because 24 subcarriers with 3.75 kHz in subframes 8–9 are allocated for an NPRACH, we can only select unit types with less than or equal to six subcarriers. The UE will transmit data in subframes 8–13 using six subcarriers.

**Figure 3:**
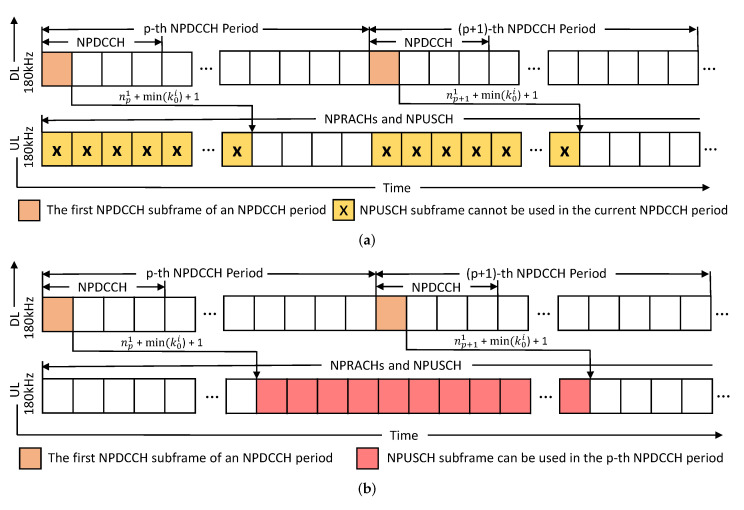
Concept of cross-cycled resource allocation. (**a**) some subframes cannot be used by the current NP; (**b**) a cell can allocate the NPUSCH subframes of the next NP, which cannot be used in the next NP, for a UE without causing wireless interference.

Because the uplink resource allocation should follow the NB-IoT protocol, as shown in Equation (1), we found that some subframes cannot be used by the current NP as shown in Figure 3a. Specifically, the first applicable NPUSCH subframe in an NP is $n_{p}^{1}+\min\left(k_{0}^{i}\right)+1$, and $n_{p}^{1}+\min\left(k_{0}^{i}\right)$ subframes will be wasted in an NP, where $\min(k_{0}^{i})$ represents the smallest scheduling delay value. The smaller the NP length, the higher is the waste percentage in an NP. To solve this problem, we propose the concept of cross-cycled resource allocation to increase resource utilization. As shown in Figure 3b, a cell can allocate the NPUSCH subframes of the next NP, which cannot be used in the next NP, for a UE without causing wireless interference. If the cell assigns a UE to transmit data using the NPUSCH subframes of the next NP (i.e., the (p+1)-th NP), the UE will stop monitoring its NPDCCH search space in the (p+1)-th NP. Because NB-IoT UEs operate in the half-duplex mode, they cannot simultaneously transmit and receive data.

### 2.2. Problem Formulation

This paper investigates the uplink resource allocation problem considering cross-cycled radio resources in NB-IoT cellular networks. This paper targets the same problem as [12], while [12] does not consider cross-cycled resource allocation. This paper attempts to minimize the consumed subframes while each UE satisfies its data requests. The system model is described as follows:

A cell must support *D* UEs. The number of NPs consumed to satisfy *D* UEs is denoted as *P*. In an uplink radio frequency, a subframe has *F* subcarriers. The frequency space of a subcarrier is *W*. There are $r_{p}^{s}$ subcarriers occupied by NPRACHs in subframe *s* in the *p*-th NP. Device *d* has an uplink data requirement with data of size $\psi_{d}$. The cell provides *U* resource unit types. Unit type *u* should use $f_{u}$ subcarriers in the frequency domain and $t_{u}$ subframes in the time domain, where $f_{u+1}>f_{u}$. The set of resource unit numbers is $I_{RU}=\left\{I^{1},I^{2},..,I^{h},..,I^{H}\right\}$ in the resource assignment field, where *H* is the maximum index and $I^{H}$ is the maximum number of resource units. We have *M* modulation and coding scheme indexes, each of which can refer to a TBS index. The cell can select a data repetition number for a UE to transmit data from the set of repetition numbers $N_{Rep}$. The cell should choose a repetition number meeting $N_{d}^{u,m}\geq {\hat{N}}_{d}^{u,m}$, $N_{d}^{u,m}\in N_{Rep}$ to guarantee the predefined transmission reliability via a success probability model (This paper can be applied no matter which success probability model is considered.), where ${\hat{N}}_{d}^{u,m}$ is the repetition requirement of UE *d* using modulation *m* and unit type *u* under an SNR value. The calculation of ${\hat{N}}_{d}^{u,m}$ values can be found in [12].

When $I_{d}^{h}$ units with type *u* using modulation *m* are assigned to UE *d*, the cell should allocate $f_{u}$ subcarriers during $I^{h}\times t_{u}\times N_{d}^{u,m}$ continuous NPUSCH subframes to UE *d* using $N_{d}^{u,m}$ repetitions. The UE can transmit a data size of $\eta (I^{h},m)$ according to the TBS table. We use an indicator function $X_{p,d}^{u,m}=1$, if the cell decides UE *d* using unit type *u* and modulation *m* in the *p*-th NP.

The parameter $R_{max}$ value is the subframe number for the NPDCCH in an NP, and the parameter *G* value is the system parameter. The length of an NP is $L=R_{max}\times G$. Both $R_{max}$ and *G* values can be used to adjust the NP length for UEs and are given in this paper. This paper considers using the DCI repetition number $R=R_{max}/8$ to transmit one DCI for each UE. The repetition number used to transmit a DCI should also meet the reliability (i.e., $R\geq {\hat{D}}_{d}$), where ${\hat{D}}_{d}$ is the required DCI repetition number of UE *d*. Specifically, when a UE needs a higher DCI repetition number, the cell should choose a higher $R_{max}$ value to increase the *R* value. Given the repetition number *R*, the DCI number is ξ (i.e., $\xi =2\times (R_{max}/R)$) in an NP, because DCI format N0 occupies one control channel element in an NPDCCH subframe, each of which has two control channel elements. We define ${\mathrm{DCI}}_{p,d}^{c}=1$ when DCI *c* in the *p*-th NP is assigned for UE *d* and 0 otherwise.

We define $S_{p}^{s}$ used to represent the subcarriers that have been assigned to UEs in subframe *s* of the *p*-th NP. $K=\{k_{0}^{1},k_{0}^{2},\ldots ,k_{0}^{i},\ldots \}$ is the set of scheduling delay values. The objective of our target problem is to decrease the total consumed subframes P×L to support *D* UEs for the uplink as much as possible. The target problem has the following constraints:

*Requirement Constraint:* Each UE *d* should transmit data size $\psi_{d}$.

(2) $$\sum_{p=1}^{P}\sum_{I^{h}\in I_{RU}}\sum_{u=1}^{U}\sum_{m=1}^{M}X_{p,d}^{u,m}\eta \left(I_{u}^{h},m\right)\geq \psi_{d},\forall d$$

*Bandwidth Constraint:* The subcarriers used by the UEs and NPRACHs cannot surpass the total subcarriers at a subframe.

(3) $$S_{p}^{s}+r_{p}^{s}\leq F,\forall s,p$$

*Signaling Constraint:* After UE *d* receives a DCI in its NPDCCH subframes, the UE can transmit data using uplink subframes.

(4) $$\begin{array}{cc} \sum_{u=1}^{U}\sum_{m=1}^{M}X_{p,d}^{u,m} & =1,\forall p,d \end{array}$$

(5) $$\begin{array}{cc} \sum_{c=1}^{\xi }{\mathrm{DCI}}_{p,d}^{c} & =1,\forall p,d \end{array}$$

The objective of this paper is formulated as:

(6) $$\underset{X_{p,d}^{u,m},{\mathrm{DCI}}_{p,d}^{c},S_{p}^{s},I_{d}^{h}}{\mathrm{minimize}}P\times L$$

with constraints (2)–(5). The notations are listed in Table 1.

## 3. Cross-NP Uplink Resource Allocation

In Section 3.1, we propose an uplink resource allocation algorithm that considers cross-cycled subframes and NPRACHs. Then, in Section 3.2, we analyze the time complexity of the proposed algorithm.

### 3.1. Algorithm Description

In this section, we propose a cross-NP uplink resource-allocation algorithm. This algorithm allocates the subframes of the current NP and uses the following NP’s uplink resources without affecting the uplink resources available to the next NP. Furthermore, this algorithm allocates UEs using the subframes that cannot be used in the next NP because of scheduling delays to increase resource utilization and use the as few subframes as possible to satisfy the needs of all UEs.

This algorithm first determines the allocation order of UEs based on the transmission spectrum efficiency. The UE with the highest spectrum efficiency is selected first. Moreover, because NPRACHs may use some subframes, this algorithm considers the number of remaining subcarriers in each subframe to select the used modulation and unit type for the UE. In addition, because the NB-IoT resource allocation protocol for the uplink should meet Equation (1), an NPUSCH start subframe is a substantial subframe. This NPUSCH start subframe occupied by a UE without efficiently using the following NPUSCH subframes will cause some subframes to never be used. Therefore, this algorithm checks whether vacating an occupied NPUSCH start subframe of a UE can release more usable subframes to serve other UEs.

The pseudo-code of the cross-NP uplink resource allocation is presented in Algorithm 1. In the first line, the number of *P* NPs to meet the data requirements of all UEs is set to 0. In line 3, we call the Transmission-Performance() function to calculate the transmission performance (bits) of each UE *d* with each unit type *u* and modulation strategy *m* in a subframe and store it in table v(d,u,m). The Transmission-Performance() function is revised from the UPLOAD-SIZE() function proposed in [12]. In Line 4, we sort the UE index in descending order according to the data requirement $\psi_{d}$ of each UE *d*. That is, we give a higher priority to the UE with a higher data requirement.

**Algorithm 1:** Cross-NP Uplink Resource Allocation

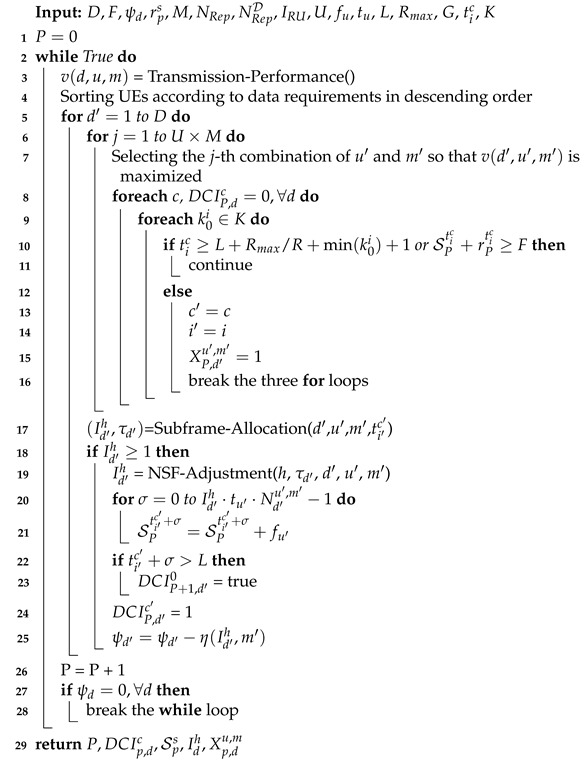



In lines 5–25, we try to schedule *D* UEs in an NP and search for a combination of the four parameters, the unit type, modulation, available DCI, and scheduling delay value, for a UE. For a selected UE $d^{\prime }$, we should select one combination of unit type *u* with modulation *m* from the UM selections (Line 6). According to table $v(d^{\prime },u,m)$, this algorithm chooses the *j*-th combination of $u^{\prime }$ and $m^{\prime }$ with the highest spectrum efficiency for UE $d^{\prime }$ (Line 7). Then, in Lines 8–16, we find a DCI *c* with a scheduling delay value $k_{0}^{i}$ for UE $d^{\prime }$. If NPUSCH start subframe $t_{i}^{c}$ is farther than the farthest unusable subframe of the next NP (i.e., $t_{i}^{c}\geq L+R_{max}/R+k_{0}^{0}+1$) or has no empty subcarriers (i.e., $S_{P}^{t_{i}^{c}}$+$r_{P}^{t_{i}^{c}}$ ≥ *F*), DCI *c* using scheduling delay $k_{0}^{i}$ is unavailable. Therefore, we try the next one (Lines 8–11). Otherwise, it indicates that the cell currently has sufficient radio resources using the four parameters for the UE. Used DCI $c^{\prime }$ and scheduling delay value index $i^{\prime }$ are set as *c* and *i*, respectively (Lines 13–14). The *j*-th combination of $u^{\prime }$ and $m^{\prime }$ can be used for the UE, and indicator $X_{P,d^{\prime }}^{u^{\prime },m^{\prime }}$ is set as 1. Because this algorithm finds four available parameters for the UE, we break the three for loops (Lines 15–16). If this algorithm cannot find a combination of the four parameters for UE $d^{\prime }$, it goes to Line 5 to serve the next UE.

In Line 17, we call the Subframe-Allocation() function to allocate subcarriers and subframes for UE $d^{\prime }$ using the four parameters. This function returns the number of allocated units for UE $d^{\prime }$ (i.e., $I_{d^{\prime }}^{h}$) and the last allocated subframe index $\tau_{d^{\prime }}$, which is designed for the NSF-Adjustment() function. When the subframe allocation is finished for UE $d^{\prime }$, if the resource assignment index *h* is larger than or equal to 1, we set the corresponding parameters and call the NSF-Adjustment() function (Lines 18–25).

The NSF-Adjustment() function is designed to reduce the number of allocated units for UE $d^{\prime }$ if a sacrificed unit from UE $d^{\prime }$ can be used to serve more UEs. When the resource assignment is adjusted, we allocate the number of $f_{u^{\prime }}$ subcarriers through continuous $I_{d^{\prime }}^{h}\cdot t_{u^{\prime }}\cdot N_{d^{\prime }}^{u^{\prime },m^{\prime }}$ subframes (Lines 20–21). If we allocate subframes of the next NP (i.e., $t_{i^{\prime }}^{c^{\prime }}+\sigma >L$) for UE $d^{\prime }$, $DCI_{P+1,d^{\prime }}^{0}$ is set as true to denote that the UE cannot be scheduled in the next NP because the UE cannot monitor its search space (Lines 22–23). Because DCI $c^{\prime }$ in the *P*-th NP is assigned to UE $d^{\prime }$, $DCI_{P,d^{\prime }}^{c^{\prime }}$ is set to 1 (Line 24), and the data requirement of UE $d^{\prime }$ is decreased by $\eta (I_{d^{\prime }}^{h},m^{\prime })$ (Line 25). When the radio resources of an NP are exhausted, or no UE should be served, the number of used NPs is increased by 1 (Line 26). Then, we check that if the data requests of all UEs are fulfilled, the algorithm is terminated, and the five parameters are returned (Lines 27–29).

The Transmission-Performance() function (Algorithm 2) calculates table v(d,u,m). In Line 2, we initialize v(d,u,m) to −∞ as unavailable, ∀d,u,m. Then, this function estimates whether UE *d* can use resource unit type *u* and modulation strategy *m* to achieve the required reliability. If the required reliability cannot be met, the number of repetitions used for transmitting data $N_{d}^{u,m}$ is −1, and we look for the next combination (Lines 6–7). Otherwise, the UE can transmit data using the unit type with the modulation, and we calculate its spectrum efficiency recorded in v(d,u,m) in Lines 8–16.

**Algorithm 2:** Transmission-Performance

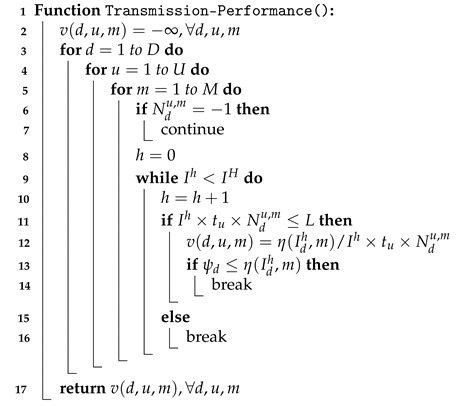



In Line 8, the resource assignment index *h* is initialized as 0. Then, the function checks whether this NP has sufficient subframes for each unit type, and the need for UE *d* is sufficient (in Lines 9–16). In every test, index *h* is increased by one until the maximum number of $I^{H}$ resource units is achieved (Lines 9–10). In Line 11, UE *d* transmitting $I^{h}$ units with type *u* and modulation strategy *m* requires $I^{h}*t_{u}*N_{d}^{u,m}$ NPUSCH subframes. Because an NP that crosses over the next NP has *L* subframes at most, $I^{h}*t_{u}*N_{d}^{u,m}\leq L$ means that UE *d* can deliver $\eta (I_{d}^{h},m)$ bits in this NP. The spectrum efficiency v(d,u,m) is $\eta \left(I_{d}^{h},m\right)/I^{h}*t_{u}*N_{d}^{u,m}$ (Lines 11–12). Otherwise, we terminate the while loop because this NP does not have sufficient resources for using this unit type and modulation (Lines 15–16). If the number of $I^{h}$ resource units can satisfy the need of UE *d*, we also terminate the while loop (Lines 13–14). When we complete v(d,u,m), this function returns v(d,u,m) in Line 17.

The Subframe-Allocation() function (Algorithm 3) calculates the number of $I_{d^{\prime }}^{h}$ units that can be continuously allocated for UE $d^{\prime }$ using unit type $u^{\prime }$ with modulation $m^{\prime }$ from NPUSCH start subframe $t_{i^{\prime }}^{c^{\prime }}$. In Line 2, variable σ is initialized to −1. σ is used to indicate the currently allocated subframe from NPUSCH start subframe $t_{i^{\prime }}^{c^{\prime }}$. Variable ϕ, initialized as 0, indicates that the number of subframes is allocated. Variable *h*, initialized to 0, is the resource assignment index (i.e., $I_{d^{\prime }}^{h}$ for UE $d^{\prime }$). In Lines 3–14, as long as the requirement $\psi_{d^{\prime }}$ of UE $d^{\prime }$ has not been met, this function will try to allocate subframes continuously one by one (i.e., σ=σ+1 in Line 4) until the maximum resource units are allocated for the UE (i.e., h<H). In Lines 5–6, if the current subframe position exceeds the NPUSCH start subframe comprising the first DCI with the minimum scheduling delay in the next NP (i.e., $L+R_{max}/R+\min\left(k_{0}^{i}\right)+1$), it means that no more subframes can be allocated in this NP, and we break the while loop. In Lines 7–8, if all the subcarriers of the subframe are occupied by NPRACHs (i.e., $r_{P}^{t_{i^{\prime }}^{c^{\prime }}+\sigma }\geq F$), we skip this subframe.

**Algorithm 3:** Subframe-Allocation

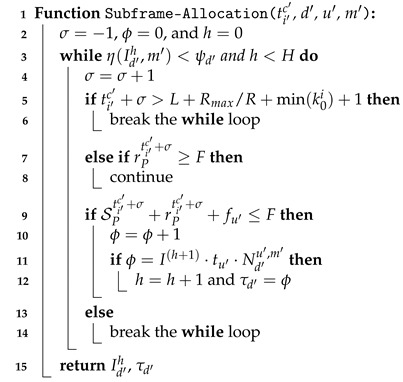



In Lines 9–10, if the unused subcarriers are sufficient for UE $d^{\prime }$ using resource $u^{\prime }$, the number of currently allocated subframes ϕ for the UE is increased by 1. Then, in Lines 11–12, we check whether the number of allocated subframes meets the number of data repetitions required for UE $d^{\prime }$ using unit type $u^{\prime }$ and modulation $m^{\prime }$. If it is satisfied, the index *h* of the resource assignment is incremented by 1, and we record the last subframe position $\tau_{d^{\prime }}$ of UE $d^{\prime }$. $\tau_{d^{\prime }}$ will be used for the NSF-Adjustment() function to increase the subframe utilization by adjusting the allocated $I_{d^{\prime }}^{h}$ units of UE $d^{\prime }$. If this subframe does not have sufficient subcarriers for the UE, this function breaks the while loop (Lines 13–14).

The NSF-Adjustment() function (algorithm 4) determines whether freeing up an occupied NPUSCH start subframe of UE $d^{\prime }$ by subtracting one resource unit to release more usable subframes to serve other UEs. In Line 2, ξ, initialized as 0, is the number of subframes that NPRACHs fully occupy. In Line 3, if the last subframe $\tau_{d^{\prime }}$ allocated to UE $d^{\prime }$ exactly is an NPUSCH start subframe and index *h* of the resource assignment for UE $d^{\prime }$ is larger than 1, we calculate the number of usable subframes when the NPUSCH start subframe is vacated in Lines 4–11. Because this paper considers cross-NP resource allocation, $\tau_{End}$ is the last allocable subframe of this NP, which can cross over the length *L* of this NP (Line 4).

**Algorithm 4:** NSF-Adjustment

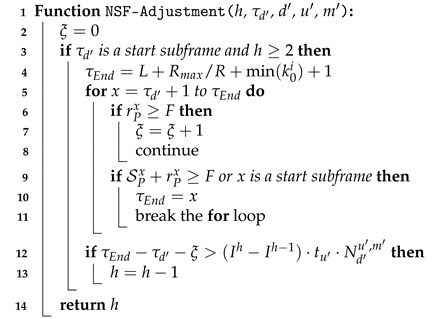



In Lines 5–11, we view each subframe to count the number of usable subframes from NPUSCH start subframe $\tau_{d^{\prime }}$ to the last allocable subframe. For each subframe *x*, if NPRACHs fully occupy the subframe, ξ is increased by 1, and we go to check the next subframe (Lines 6–8). If the available subcarriers of the subframe are all allocated to UEs or the subframe is another NPUSCH start subframe, the last allocable subframe is updated as the current subframe *x*, and we break the for loop (Lines 9–11). In Line 9, the first condition is because we should ensure that the allocable subframes are continuous. The second condition is that an NPUSCH start subframe is a vital subframe, which should be reserved for another UE. In Lines 12–13, if the released subframes (i.e., $\tau_{End}-\tau_{d^{\prime }}-\xi$) by vacating the NPUSCH start subframe are greater than the sacrificing subframes from UE $d^{\prime }$, we decrease the number of resource units allocated to UE $d^{\prime }$ and index *h* is decreased by 1.

### 3.2. Property of the Proposed Algorithm

**Theorem** **1.** *The time complexity of Algorithm 1 is $O(DUM+UM\xi^{2}|K|+L)$ for an NP.*

**Proof.**  In Algorithm 1, the Transmission-Performance() function takes O(DUM) time to calculate the number of bits that each UE can transmit using different unit types and modulations. For a UE, Algorithm 1 selects a combination of unit type and modulation from UM selections. Then, Algorithm 1 finds sufficient subcarriers and subframes for a combination by looking for each DCI and scheduling delay. Because at most ξ UEs can be allocated in an NP, the process of Lines 5–16 takes $O(UM\xi^{2}|K|)$ time. Then, because there are at most *L* subframes, the Subframe-Allocation() function and setting the corresponding parameters take O(L) time. Thus, the algorithm’s time complexity is $O(DUM+UM\xi^{2}|K|+L)$. □

## 4. Performance Evaluation

### 4.1. Simulation Setups

The simulation setups are based on 3GPP specifications [1,14]. We compare the performance of the proposed algorithm (*cross-NP uplink resource allocation (CNPURA)*) with two baselines. The first baseline is the *link adaptation and uplink resource allocation* (LAURA) algorithm [12]. The *LAURA* algorithm first uses a dynamic programming algorithm to determine which UEs should be allocated in an NP and decide the used unit type and modulation scheme for each chosen UE without considering NPRACHs. The dynamic programming algorithm can maximize the transmission bits in an NP. Then, *LAURA* considers NPRACHs to adjust the used unit type, modulation, and allocated uplink radio resources for each scheduled UE. The second baseline is *energy saving (ES)* [11]. The *ES* algorithm schedules the UE in order from having the fewest usable combinations of unit types and modulations to the most usable pairs. Then, because the algorithm considers energy efficiency, it picks the unit type with the modulation for the currently scheduled UE requiring the fewest subframes for transmitting data. Because the *ES* scheme does not consider uplink resource allocation, it adopts the two functions for the uplink resource allocation of the *LAURA* algorithm to find DCIs and delay values and to decide the resource assignment for each UE.

We use MATLAB to build our simulation. The simulation considers a cell serving UEs varying from 400 to 2000. The transmission power of the cell and each UE are set to 32 and 23 dBm, respectively [11]. The distance φ of each UE from the cell is distributed randomly between [1, 5000] meters, considering only one CE level. The demand of each UE is randomly set from 20 to 200 bytes [8]. Our channel model considers the path loss model as 120.9+30.76log(θ) dB, where the unit of θ is kilometers [11]. The thermal noise density is −173 dBm/Hz. There are four types of resource unit. The SNR of each UE can be estimated using the path loss model, unit type, and distance φ of the UE from the cell.

The set of resource unit numbers that can be assigned to a UE in an NP is $I_{RU}=\left\{1,2,3,4,5,6,8,10\right\}$ [1]. There are 14 modulation and coding scheme indices. The uplink TBS, comprising the adopted modulation-coding scheme index and the assigned resource unit number, is shown in Table 2. The selectable $R_{max}$ values are 1, 2, 4, 8, 16, 32, 64, 128, 256, 512, 1024, and 2048. The selectable *G* values are 1.5, 2, 4, 8, 16, 32, 48, and 64. The DCI repetition number is set as $R_{max}/8$ for all the UEs. The scheduling delay values for the uplink are 8, 16, 32, and 64. The repetition number for a UE to deliver data can be chosen from: 1, 2, 4, 8, 16, 32, 64, or 128 [1]. The selected $R_{max}$ and *G* values should satisfy the DCI and data repetition requirements of all UEs. In the simulation, we consider three different NPRACHs configured according to [12].

### 4.2. Simulation Results

**Figure 4:**
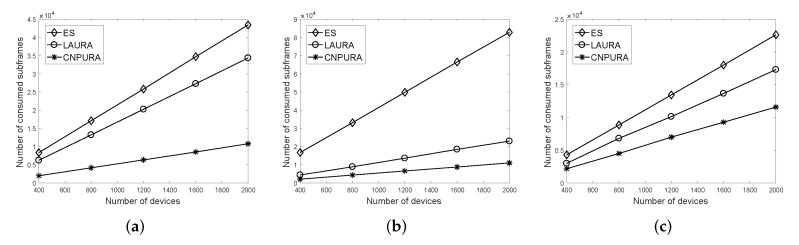
Effect of the UE numbers on the number of consumed subframes under different $R_{max}$ and *G* values. (**a**) $R_{max}=8$ and G=2. (**b**) $R_{max}=16$ and G=1.5. (**c**) $R_{max}=16$ and G=2.

Figure 4 shows the effect of adjusting the number of UEs on the consumption of subframes under the three combinations of $R_{max}$ and *G* values. Because more UEs will have more data requirements, the consumption of NPUSCH subframes also increases as the number of UEs increases. Compared with the three different combinations of $R_{max}$ and *G* values, the performance improvement of the proposed *CNPURA* is more evident under $R_{max}=8$ and G=2. When the NP length is shorter, a higher percentage of subframes in an NP (i.e., $\frac{R_{max}}{R_{max}\times G}$%) cannot be used under the *ES* and *LAURA* algorithms. *CNPURA* can efficiently use the NPUSCH subframes of the next NP to increase the subframe utilization, especially when more NPUSCH start subframes reside in the next NP. The proposed algorithm can respectively outperform *LAURA* about 69%, 53%, and 32% in Figure 4a–c, in terms of the consumed subframes. Compared with *ES*, *CNPURA* can obviously reduce more subframe consumption.

**Figure 5:**
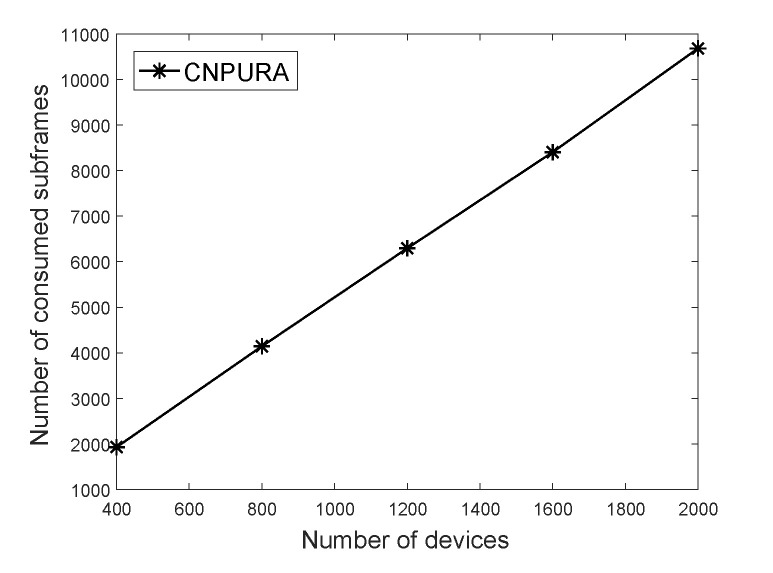
Effect of the UE numbers on the number of consumed subframes under $R_{max}=8$ and G=1.5.

**Figure 6:**
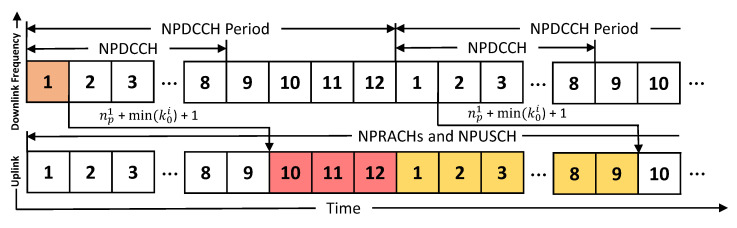
An illustration for using the subframes of the next NP.

The proposed algorithm not only consumes fewer subframes but can also be adopted for more $R_{max}$ and *G* values. When $R_{max}=8$ and G=1.5, Figure 5 only shows our proposed *CNPURA*, because the *ES* and *LAURA* algorithms are not available in this case while the proposed algorithm is still workable. This is because the two baselines cannot meet the data repetition requirements of all the UEs and are explained in detail in Figure 6.

In Figure 6, because the two baselines do not consider cross-cycled resource allocation to use subframes of the next NP, they can only use three subframes (i.e., subframes 10–12 marked by red color) in an NP, which are not sufficient to satisfy the data repetition requirements of all UEs. By contrast, our proposed algorithm can utilize a total of 12 subframes marked by the red and yellow colors, where subframes 1–9 cannot be used by the next NP limited to the NB-IoT resource allocation protocol. Therefore, the proposed algorithm can be flexibly applied for more $R_{max}$ and *G* values, especially for smaller $R_{max}$ and *G* values. Resource allocation algorithms operating under smaller $R_{max}$ and *G* values generally can consume fewer subframes. The number of consumed subframes in Figure 5 is slightly lower than that in Figure 4a.

**Figure 7:**
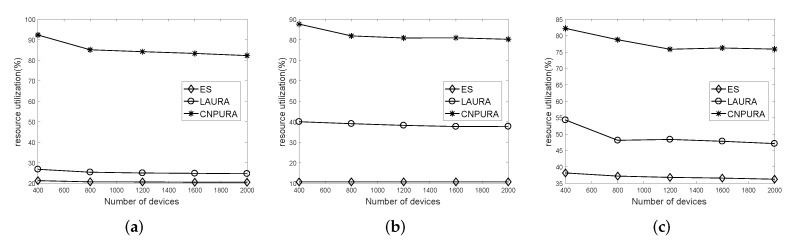
Effect of UE numbers on the resource utilization under different $R_{max}$ and *G* values. (**a**) $R_{max}=8$ and G=2. (**b**) $R_{max}=16$ and G=1.5. (**c**) $R_{max}=16$ and G=2.

Figure 7 shows the impacts of adjusting the number of UEs on the resource utilization under different $R_{max}$ and *G* values. Resource utilization is defined as the number of consumed subcarriers divided by the total number of subcarriers. For the three combinations of $R_{max}$ and *G* values, as the number of UEs increases, the resource utilization will slightly decrease because more UEs should compete for the limited radio resources. Figure 7 demonstrates that the proposed algorithm considers cross-cycled resource allocation so that the resource utilization is always higher than 80%, while the resource utilization of the *ES* and *LAURA* algorithms is lower than 30%, 40%, and 55% in Figure 7a–c, respectively. The two baselines waste a proportion of the subframes in each NP, resulting in lower resource utilization. Moreover, the waste proportion will be higher when the NP length is shorter.

**Figure 8:**
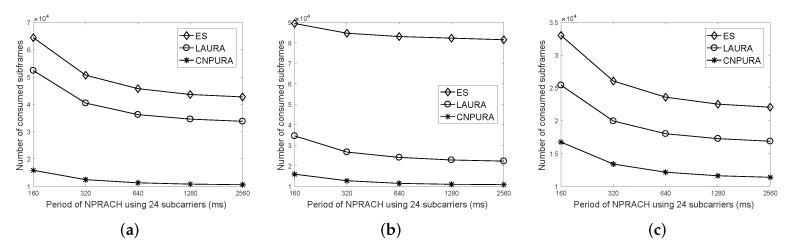
Effect of the NPRACH periods on the number of consumed subframes under different $R_{max}$ and *G* values with 2000 UEs. (**a**) $R_{max}=8$ and G=2. (**b**) $R_{max}=16$ and G=1.5. (**c**) $R_{max}=16$ and G=2.

Figure 8 shows the effect of different NPRACH cycles using 24 subcarriers on the subframe consumption under different $R_{max}$ and *G* values with 2000 UEs. When the NPRACH cycle is longer, we have more NPUSCH subframes. Therefore, the three methods can diminish the consumed subframes further. We can observe that the change in NPRACH cycles has a lower impact on the subframe consumption under our proposed algorithm than under *LAURA* and *ES*. This is because the proposed algorithm considers NPRACHs to adjust the unit type and modulation scheme and efficiently uses the subframes of the next NP. Therefore, the proposed algorithm can have more choices of unit types and modulation schemes for each UE. By contrast, although *LAURA* also considers NPRACHs to select the used unit type and modulation scheme for each UE, it only considers using the subframes of the current NP, which limits the selection of unit types and modulation schemes. The simulation results justify our motivation that cross-cycled resource allocation is important for achieving massive connections and for increasing resource utilization, especially for the UEs with better channel qualities.

## 5. Conclusions

This paper investigated the cross-cycled uplink resource allocation problem considering the use of radio resources of the next NP over NB-IoT. Our optimization problem minimizes the consumed subframes for satisfying the data requirement of each UE. To overcome this issue, we propose a cross-cycled uplink resource-allocation algorithm. The algorithm considers using the subframes that cannot be used in the next NP without causing interference for some UEs. Moreover, the proposed algorithm considers NPRACHs to adjust the modulation and unit type for each UE according to the remaining subcarriers. The simulation results justify that cross-cycled uplink resource allocation significantly increases resource utilization and is essential for NB-IoT, especially for UEs with high channel qualities. Compared with the two baselines, the proposed algorithm can not only be applied for a higher number of $R_{max}$ and *G* values but can also significantly decrease the consumed subframes. The performance improvement is more evident as $R_{max}$ and *G* values become small.

## Figures and Tables

**Table 1:** Notations.

Symbol	Depiction
*D*	The number of UEs
*P*	The consumed NPDCCH periods
*F*	The subcarriers in a subframe
*M*	The number of modulation and coding indexes
$\psi_{d}$	UE *d*’s data requirement
${\hat{N}}_{d}^{u,m}$	The data repetition requirement of UE *d* as unit type *u* and modulation *m* is adopted
$N_{Rep}$	The set of repetition numbers for delivering data
$N_{Rep}^{D}$	The set of repetition numbers for delivering a DCI
$I_{RU}$	The set of resource unit numbers
*U*	The number of different unit types
$\eta (I^{h},m)$	The TBS as $I^{h}$ resource units with modulation *m* is transmitted.
$f_{u}$	The subcarriers occupied by unit type *u*
$r_{p}^{s}$	The subcarriers used by NPRACHs in subframe *s* of the *p*-th NP
$t_{u}$	The subframes needed by unit type *u*
*L*	The length of an NP
$R_{max}$	The number of NPDCCH subframes
*G*	The system parameter for determining the length of NPDSCH in an NPDCCH period
*R*	The repetitions using for delivering one DCI
$n_{p}^{c}$	The last subframe in a series of NPDCCH subframes for delivering DCI *c* in the *p*-th NP
${\mathrm{DCI}}_{p,d}^{c}$	The function records whether DCI *c* of the *p*-th NP is assigned for UE *d* or not
${\hat{D}}_{d}$	The DCI repetition requirement of UE *d*
*K*	The set of scheduling delays
$t_{i}^{c}$	The NPUSCH start subframe as DCI *c* with delay $k_{0}^{i}$
$S_{p}^{s}$	The subcarriers assigned for UEs in subframe *s* of the *p*-th NP.
$X_{p,d}^{u,m}$	The function records whether the cell decides UE *d* using unit type *u* and modulation *m* in the *p*-th NP or not

**Table 2:** TBS table for NPUSCH, $\eta (I^{h},m)$.

*m*	$I^{h}$							
	1	2	3	4	5	6	8	10
1	16	32	56	88	120	152	208	256
2	24	56	88	144	176	208	256	344
3	32	72	144	176	208	256	328	424
4	40	104	176	208	256	328	440	568
5	56	120	208	256	328	408	552	680
6	72	144	224	328	424	504	680	872
7	88	176	256	392	504	600	808	1032
8	104	224	328	472	584	680	968	1224
9	120	256	392	536	680	808	1096	1352
10	136	296	456	616	776	936	1256	1544
11	144	328	504	680	872	1032	1384	1736
12	176	376	584	776	1000	1192	1608	2024
13	208	440	680	1000	1128	1352	1800	2280
14	224	488	744	1032	1256	1544	2024	2536

## Data Availability

The data used to support the findings of this paper are available upon request.

## Abbreviations

The following abbreviations are used in this manuscript:

NB-IoT	Narrowband Internet of Things
NPUSCH	Narrowband physical uplink shared channel
UE	User equipment
NPDCCH	Narrowband physical downlink control channel
NPDSCH	Narrowband physical downlink shared channel
DCI	Downlink control indicator
3GPP	3rd generation partnership project
NP	NPDCCH period
DL	Downlink frequency
UL	Uplink frequency
CE	Coverage enhancement
NPRACH	Narrowband physical random access channel
OFDMA	Orthogonal frequency division multiple access
SNR	Signal-to-noise ratio
TBS	Transport block size

## References

[1] 3GPP TS 36.213 V16.0.0 Technical Specification Group Radio Access Network; Evolved Universal Terrestrial Radio Access (E-UTRA); Physical layer procedures. https://portal.3gpp.org/desktopmodules/Specifications/SpecificationDetails.aspx?specificationId=2427.

[2] Cisco (2020). Cisco Visual Networking Index: Global Mobile Data Traffic Forecast Update, 2018–2023. http://www.cisco.com/.

[3] Ericsson (2020). Ericsson Mobility Report. https://www.ericsson.com/en/mobility-report/reports.

[4] Yu C., Yu L., Wu Y., He Y., Lu Q. (2017). Uplink Scheduling and Link Adaptation for Narrowband Internet of Things Systems. IEEE Access.

[5] Elgarhy O., Reggiani L., Malik H., Alam M.M., Imran M.A. (2021). Rate-Latency Optimization for NB-IoT With Adaptive Resource Unit Configuration in Uplink Transmission. IEEE Syst. J..

[6] Hsieh B.Z., Chao Y.H., Cheng R.G., Nikaein N. Design of a UE-specific Uplink Scheduler for Narrowband Internet-of-Things (NB-IoT) Systems. Proceedings of the IEEE International Conference on Intelligent Green Building and Smart Grid (IGBSG).

[7] Mostafa A.E., Zhou Y., Wong V.W. (2019). Connection Density Maximization of Narrowband IoT Systems With NOMA. IEEE Trans. Wirel. Commun..

[8] Huang C.W., Tseng S.C., Lin P., Kawamoto Y. (2019). Radio Resource Scheduling for Narrowband Internet of Things Systems: A Performance Study. IEEE Netw..

[9] Pei E., Wang Z., Li Y. An Adaptive Uplink Resource Allocation Algorithm in NB-IoT. Proceedings of the IEEE Vehicular Technology Conference (VTC2021-Spring).

[10] Kodheli O., Maturo N., Chatzinotas S., Andrenacci S., Zimmer F. (2021). NB-IoT via LEO Satellites: An Efficient Resource Allocation Strategy for Uplink Data Transmission. IEEE Internet Things J..

[11] Liang J.M., Wu K.R., Chen J.J., Liu P.Y., Tseng Y.C. (2018). Energy-Efficient Uplink Resource Units Scheduling for Ultra-Reliable Communications in NB-IoT Networks. Wirel. Commun. Mob. Comput..

[12] Yu Y.J., Wang J.K. (2021). NPRACH-Aware Link Adaptation and Uplink Resource Allocation in NB-IoT Cellular Networks. IEEE Trans. Veh. Technol..

[13] Yu Y.J. (2021). NPDCCH Period Adaptation and Downlink Scheduling for NB-IoT Networks. IEEE Internet Things J..

[14] 3GPP TS 36.331 V16.0.0 Technical Specification Group Radio Access Network; Evolved Universal Terrestrial Radio Access (E-UTRA); Radio Resource Control (RRC); Protocol specification. https://portal.3gpp.org/desktopmodules/Specifications/SpecificationDetails.aspx?specificationId=2440.

